# Allostatic load and subsequent all-cause mortality: which biological markers drive the relationship? Findings from a UK birth cohort

**DOI:** 10.1007/s10654-018-0364-1

**Published:** 2018-02-23

**Authors:** Raphaële Castagné, Valérie Garès, Maryam Karimi, Marc Chadeau-Hyam, Paolo Vineis, Cyrille Delpierre, Michelle Kelly-Irving, for the Lifepath Consortium, Harri Alenius, Mauricio Avendano, Valeria Baltar, Mel Bartley, Henrique Barros, Murielle Bochud, Cristian Carmeli, Luca Carra, Giuseppe Costa, Emilie Courtin, Angela Donkin, Angelo D’Errico, Pierre-Antoine Dugue, Paul Elliott, Giovanni Fiorito, Silvia Fraga, Martina Gandini, Graham Giles, Marcel Goldberg, Dario Greco, Allison Hodge, Piia Karisola, Mika Kivimaki, Jessica Laine, Thierry Lang, Richard Layte, Benoit Lepage, Johan Mackenbach, Michael Marmot, Carlos de Mestral, Cathal McCrory, Roger Milne, Peter Muennig, Wilma Nusselder, Dusan Petrovic, Silvia Polidoro, Martin Preisig, Olli Raitakari, Ana Isabel Ribeiro, Fulvio Ricceri, Erica Reinhard, Oliver Robinson, Jose Rubio Valverde, Roberto Satolli, Gianluca Severi, Silvia Stringhini, Joannie Tieulent, Salvatore Vaccarella, Anne-Claire Vergnaud, Peter Vollenweider, Marie Zins

**Affiliations:** 1 Faculty of Medicine Purpan, LEASP UMR 1027, Inserm-Université Toulouse III Paul Sabatier, 37 Allées Jules Guesde, 31000 Toulouse, France; 2 Department of Epidemiology and Biostatistics, MRC-PHE Centre for Environment and Health, School of Public Health, Faculty of Medicine, Imperial College, Norfolk Place, London, W2 1PG UK; 3 Molecular and Genetic Epidemiology Unit, Italian Institute for Genomic Medicine (IIGM), Via Nizza 52, 10126 Turin, Italy

**Keywords:** Allostatic load, Mortality, Cohort study, Social environment, Health behaviours

## Abstract

The concept of allostatic load (AL) refers to the idea of a global physiological ‘wear and tear’ resulting from the adaptation to the environment through the stress response systems over the life span. The link between socioeconomic position (SEP) and mortality has now been established, and there is evidence that AL may capture the link between SEP and mortality. In order to quantitatively assess the role of AL on mortality, we use data from the 1958 British birth cohort including eleven year mortality in 8,113 adults. Specifically, we interrogate the hypothesis of a cumulative biological risk (allostatic load) reflecting 4 physiological systems potentially predicting future risk of death (N = 132). AL was defined using 14 biomarkers assayed in blood from a biosample collected at 44 years of age. Cox proportional hazard regression analysis revealed that higher allostatic load at 44 years old was a significant predictor of mortality 11 years later [HR = 3.56 (2.3 to 5.53)]. We found that this relationship was not solely related to early-life SEP, adverse childhood experiences and young adulthood health status, behaviours and SEP [HR = 2.57 (1.59 to 4.15)]. Regarding the ability of each physiological system and biomarkers to predict future death, our results suggest that the cumulative measure was advantageous compared to evaluating each physiological system sub-score and biomarker separately. Our findings add some evidence of a biological embodiment in response to stress which ultimately affects mortality.

**Electronic supplementary material:**

The online version of this article (10.1007/s10654-018-0364-1) contains supplementary material, which is available to authorized users.

## Introduction

Health inequalities have been highlighted between socioeconomic groups within populations worldwide [1, 2]. In particular, the rate of premature mortality, is found higher among people with more disadvantaged socioeconomic positions (SEP) across countries and at all stages of the lifespan [2–6]. Several epidemiological studies have shown that behaviours and lifestyle factors are important determinants of mortality, but do not fully explain the social gradient [7, 8]. A better understanding of the aetiological pathways through which adverse health outcomes are generated is key to alleviate the effects of social inequalities in health.

Persistent socioeconomic disadvantage, and psychosocial adversities across the life course have been linked to chronic over activation of stress response mechanisms. The neuroendocrine hormones release and the ensuing biochemical cascade build up over time due to these chronic exposures, promoting the initiation, development and progression of disease [9–11]. The prolonged activation of compensatory physiological mechanisms can lead to a physiological ‘wear-and-tear’, termed allostatic load (AL) [9, 11–13]. The AL model of chronic stress focuses on glucocorticoid dysregulation as part of a ‘network of allostasis’ involving autonomic, endocrine, metabolic, and immune mediators [14, 15]. A variety of studies using measures of AL have suggested its association with numerous health outcomes and higher all-cause of mortality risk [16].

Embodiment refers to how people incorporate, biologically, the world in which they live, including social and ecological circumstances [17]. The relationship between life course SEP and chronic stress exemplifies the embodiment dynamics through its complexity and the pathways (behavioural, material and psychosocial) through which SEP can affect health in later life [18]. Three broad, complementary and connected pathways across the life course have been outlined as the main routes towards health inequalities. These involve i)“personal” factors such as health behaviours/lifestyle, ii) structural factors including material resources and socio-economic conditions and iii) psychosocial processes involving physiological responses to environmental stimuli through a cascade of information-processing pathways in the central nervous system leading to a series of bio-physiological adjustments [18, 19].

Multiple studies have examined the relationship between the social environment and AL, showing that AL was socially distributed [16]. Gustafsson et al. [20] examined the influence of life course SEP from 16 years of age on AL observing that cumulative socioeconomic disadvantages was related to AL, in a northern Swedish cohort with 27 years of follow-up data. Regarding pathways, they reported that social adversity during adolescence for women and during early adulthood for men was associated with later AL independently of health behaviours and adulthood adversities [21].

Our own previous studies on the 1958 National Child Development Study (NCDS) evaluated the contribution and the relationship between these three broad pathways (“personal”, “structural” and psychosocial pathways) on all-cause mortality and cancer incidence in a longitudinal setting. To better characterise the social-to-biological embodiment processes, we assessed the contribution of the three main pathways over the life course in mediating the association between childhood SEP and AL. Our results highlighted that the relationship between childhood SEP and AL in early adulthood was mediated by an educational, material and health behaviours pathway [22]. We also examined how exposure to adverse childhood experiences (ACEs) was associated with a higher AL score in midlife, a relationship which was largely explained by early adult behavioural and socioeconomic factors [23]. Having a higher AL at 44 years old was also associated with poorer subjective health five years later using a latent health variable derived from sleep patterns, physical and mental health at 50 years old, after adjusting for life course SEP and behaviours [24]. Additional studies are required to gain understanding on the relationships between AL, its components and a variety of health outcomes.

This study aims to investigate the relationship between AL and subsequent mortality risk in a large prospective birth cohort. We use data from 14 blood biomarkers representing four physiological systems (neuroendocrine, immune and inflammatory, metabolic, cardiovascular system) measured in 45 years old adults. We first examine the performance of the AL score in predicting mortality up to 11 years after blood collection, using models correcting for a priori life course confounders. In a second step we also evaluate the relative contribution of each physiological systems, and each individual biomarkers entering in the AL score definition, in the relationship linking AL and death.

## Materials and methods

### Study design and participants

We used data from the 1958 National Child Development Study (NCDS), which includes all live births during 1 week in 1958 (n = 18,555) in Great Britain. The NCDS has been described in detail elsewhere [25]. Information on health, economic, social and developmental factors has been collected from participants at ages 7, 11, 16, 23, 33, 42, 44/45, 46, 50 and 55 years. Ethical approval has been obtained for this study and study participants have provided informed consent. The survey at age 44/45 years was a biomedical survey on a subsample of participants (n = 9,377) during which blood samples were collected [26]. Venous blood samples were obtained without prior fasting and posted to the collaborating laboratory. The 45-year biomedical survey was approved by the South-East Multi-Centre Research Ethics Committee, and written consent for use of information in medical research studies was obtained from the participants. Participants in this survey were found representative of the general cohort [27]. Based on their representative nature and on the availability of biomarkers we used these participants to evaluate the allostatic load. A set of 1,264 participants was excluded from our analyses including pregnant women and those for whom blood was not obtained. Additionally, individuals who died before blood sample collection (N = 1,251) or those who died with missing biological data were excluded (N = 234) leaving 132 deaths for 7,981 living participants.

### All-cause mortality

Deaths were ascertained systematically by the Centre for Longitudinal Studies upon receipt of death certificates from the National Health Service Central Register (NHSCR). The mortality data most recently available to researchers provided information on date of death up to December 2013. Since information on death was obtained from the NHSCR, even when individuals were lost to follow-up in the cohort, information on their death will have been received. The follow-up time was calculated from the date of blood collection to the date of death for participants who died and up to 1 December 2013 for survivors.

### Allostatic load

Allostatic load was defined consistently with previous work using the NCDS and in accordance with the initial definition of AL [28] : among available biomarkers, 14 were used representing four physiological systems: the neuroendocrine system [salivary cortisol t1, salivary cortisol t1–t2]; the immune and inflammatory system [insulin-like growth factor-1 (IGF1), C-reactive protein (CRP), fibrinogen, Immunoglobulin E (IgE)]; the metabolic system [high-density lipoprotein (HDL), low-density lipoprotein (LDL), triglycerides, glycosylated haemoglobin (HbA1C)]; the cardiovascular and respiratory systems: [systolic blood pressure (SBP), diastolic blood pressure (DBP), heart rate, peak expiratory flow]. Each biomarker was then dichotomized into high risk versus low risk according to sex-specific quartiles. The high-risk quartile was the highest quartile of all biomarkers, except for those found inversely related to health outcomes (HDL, salivary cortisol t1–t2, IGF1, peak expiratory flow). Descriptive information and high-risk cut-off values are reported in Supplementary Table 1. The AL score was calculated by summing the 14 dichotomous scores for each of the 14 markers. We considered a 3 group variable based on tertiles in the total population contrasting individuals with ‘low [0:2]’, ‘mid [3:4]’, and ‘high [5:12]’ AL. A conservative approach (maximum bias) was used for the individuals with missing data for each biomarker, systematically considering them to be not at risk for the missing biomarker. In the study population, 26 individuals had missing data on all biomarkers and were therefore excluded from the analyses.

### Physiological system sub-scores & individual components

For each of the four physiological systems, we calculated a sub-score by summing the dichotomized marker involved in each system. To compare the shape and magnitude of the associations, physiological sub-scores were categorized into 3 groups ‘low [0]’, ‘mid [1]’, ‘high [2:4]’ when the number of biomarkers within each system was ≥ 2 (immune and inflammatory, metabolic and cardiovascular system) and in 2 groups otherwise (neuroendocrine system; ‘low [0]’ vs ‘high [1, 2]’). We further considered all dichotomized markers separately. We corrected for multiple testing using a Bonferroni corrected significance level α’ = 0.05/5 = 0.01 when testing the 5 sub-scores separately, and α’ = 0.05/14 = 0.003 when testing the 14 markers individually.

### Covariates

Key variables that could act as a confounders in the association linking AL and subsequent death, were selected a priori based on our previous works [22–24, 29] and can be categorised in three main categories according to the life stages they relate to: early-life and childhood, young adulthood, and adulthood. A detailed description of covariates is reported elsewhere [23, 29] and so a brief description follows (Supplementary Table 2).

*Early life and Childhood Risk Factors* variables that were likely to be social or biological confounders were selected from a questionnaire completed at birth by the cohort member’s mother: mother’s education level, mother’s partner’s (or mother’s father’s if unavailable) social class, overcrowded household, maternal smoking during pregnancy, mother’s body mass index (BMI). Gender and birth weight were also included. A binary childhood pathologies variable was constructed using data collected at ages 7, 11, and 16 y based on both maternal reporting and medical examinations including congenital conditions, moderate/severe disabilities, chronic respiratory or circulatory conditions, sensory impairments, and special schooling. Adverse childhood experiences (ACEs) were defined as intra-familial events or conditions causing chronic stress responses in the child’s immediate environment. ACEs were measured by counting the reports of: child in care, physical neglect, offenders, parental separation, mental illness and alcohol abuse. A three category variable was then constructed (0 adversity/1 adversity/more than one adversity).

*Young adulthood risk factors* included the respondent’s educational attainment at 23 y (A level/O level/no qualification) and a binary indicator of psychological malaise which was set to true if the participant reported to experience more than 7 (out of 24) symptoms of a “malaise inventory” assessing symptoms of depression and/or anxiety.

*Adult risk factors* included self-reported physical activity, alcohol consumption, smoking status, BMI, own occupational class and housing tenure.

### Statistical analysis

*Descriptive analyses* Effectives, expressed as percentages, for categorical covariates were used to describe baseline characteristics of the 132 deaths with biological data and of the 234 deaths without blood collection. Selected characteristics of the population were also presented in the total population and by death status on non-imputed data. To determine significant associations and differences, we used Chi square statistics of independence and t-tests as appropriate.

*Cox regression* Multivariate Cox proportional hazards regression was used to estimate hazard ratios (HR) and 95% confidence intervals (CI) for the association between AL, biological sub-scores and individual biomarkers with mortality. Cox models were used to compute the HRs for the participants in the intermediate and high-risk group as compared to those in the low-risk group for each biological variable (AL, sub-scores and individual biomarkers) using follow-up time was used as the time scale. The overall associations between AL with all-cause mortality were adjusted for sex. Models were then chronologically adjusted for the *early life, childhood and young adulthood* confounders (model 1) and additionally controlled for *adulthood* confounders (model 2).

Using the non-imputed data, we tested the proportional hazards assumption for the allostatic load. Both statistical testing (using Schoenfeld residuals) and visual inspection (scatterplots and smoothed plots of scaled Schoenfeld residuals vs. time; ‘log–log’ plots) were performed and showed no violation of the assumption of proportional risk. Kaplan–Meier curves were also constructed for participants with low, intermediate and high AL. Cumulative probability of deaths were constructed for participants with in the high risk group for each biological variable (AL, sub-scores and individuals biomarkers) with the use of the Kaplan–Meier method.

*Missing data* To control for possible bias due to missing data, we imputed data for covariates with missing data using the multiple imputation in the overall population (N = 18,558) using the MICE R package [30]. Twenty imputations were conducted taking the missing-at-random assumption. Each variable with missing values was imputed including all covariates used in the models as well as variables from other sweeps correlated with the variable to impute, but excluding the AL and death outcome (Supplementary Table 2). To obtain Cox regression estimates from the multiply imputed data, Rubin’s combination rules were used.

*Sensitivity analyses* We conducted three different sensitivity analyses. The first sensitivity analysis was to ensure that our results were not biased by imputing the missing values; we ran our analyses on the non-imputed dataset. To take into account potential gender differences, multivariate cox proportional hazards regression were run in men and women separately. Finally to evaluate the stability of the AL score, we ran a series of model 2 multivariate cox proportional hazards regressions controlling for each sub-score and each biomarkers individually.

Statistical analyses were performed in R v3.4.0 [31] using the RStudio environment v1.0.143.

## Results

We first compared the subsample of 132 deaths with biological data included in our analyses from the 234 deaths without blood collection. Individuals in each group did not differ in terms of adult covariates (Supplementary Table 3). However, participants without blood samples were more likely to come from overcrowded households (24.3% vs 11.11%, p = 0.005) and their mothers were more likely to have left school before 14 years old (81.2% vs 72.4%, p = 0.079, Supplementary Table 3). Table 1 presents the distribution of selected characteristics for NCDS participants according to death status and for the overall sample. During a median follow-up time of 10.5 years (10–90 percentile: 10.0–11.1), 132 participants with blood collected in the cohort died. Compared with participants who did not die (N = 7,981), those who died (N = 132) were more likely to be men; to have suffered from childhood pathologies; their fathers were more likely to have had a ‘manual’ occupation; their mothers to have been smokers during pregnancy; and they were more likely to have a low birthweight. They also had fewer qualifications; were more likely to have a manual occupation as adults and to rent their accommodation. Participants who died were also more inactive and likely to smoke.

**Table 1:** Descriptive statistics of selected sociodemographic, health-related and biological characteristics on the NCDS subsample by mortality status

Characteristics	No death (N = 7981)	Death (N = 132)	*P*-value	Total (N = 8113)
Gender	n = 7981	n = 132	0.028	n = 8113
Men, n(%)	3978 (49.84)	79 (59.85)		4057 (50.01)
Women, n(%)	4003 (50.16)	53 (40.15)		4056 (49.99)
Mother’s education level	n = 7513	n = 127	0.916	n = 7640
Left school at 15 or later, n(%)	2009 (26.74)	35 (27.56)		2044 (26.75)
Left school before 14, n(%)	5504 (73.26)	92 (72.44)		5596 (73.25)
Father’s social class at birth	n = 7214	n = 122	0.007	n = 7336
Non manual, n(%)	2134 (29.58)	22 (18.03)		2156 (29.39)
Manual, n(%)	5080 (70.42)	100 (81.97)		5180 (70.61)
Overcrowding	n = 7359	n = 126	0.897	n = 7485
> 1.5 people per room, n(%)	875 (11.89)	14 (11.11)		889 (11.88)
< 1.5 people per room, n(%)	6484 (88.11)	112 (88.89)		6596 (88.12)
Mother’s BMI	n = 7150	n = 124	0.339	n = 7274
Normal, n(%)	5188 (72.56)	84 (67.74)		5272 (72.48)
Underweight, n(%)	318 (4.45)	4 (3.23)		322 (4.43)
Overweight, n(%)	1354 (18.94)	28 (22.58)		1382 (19)
Obese, n(%)	290 (4.06)	8 (6.45)		298 (4.1)
Mother smoked during pregnancy	n = 7467	n = 128	0.021	n = 7595
No, n(%)	5056 (67.71)	73 (57.03)		5129 (67.53)
Sometimes, n(%)	442 (5.92)	9 (7.03)		451 (5.94)
Moderately, n(%)	1116 (14.95)	21 (16.41)		1137 (14.97)
Heavily, n(%)	853 (11.42)	25 (19.53)		878 (11.56)
Birth weight	n = 7331	n = 123	0.065	n = 7454
Q1: low weight, n(%)	1634 (22.29)	38 (30.89)		1672 (22.43)
Q2, n(%)	2030 (27.69)	37 (30.08)		2067 (27.73)
Q3, n(%)	1892 (25.81)	24 (19.51)		1916 (25.7)
Q4: high weight, n(%)	1775 (24.21)	24 (19.51)		1799 (24.13)
Childhood pathologies	n = 7928	n = 131	0.01	n = 8059
No, n(%)	6004 (75.73)	86 (65.65)		6090 (75.57)
Yes, n(%)	1924 (24.27)	45 (34.35)		1969 (24.43)
Adverse Childhood Experiences	n = 7407	n = 128	0.002	n = 7535
None, n(%)	5412 (73.07)	84 (65.62)		5496 (72.94)
One, n(%)	1524 (20.58)	26 (20.31)		1550 (20.57)
Two or more, n(%)	471 (6.36)	18 (14.06)		489 (6.49)
Malaise inventory at 23	n = 6900	n = 109	0.051	n = 7009
No, n(%)	6436 (93.28)	96 (88.07)		6532 (93.19)
Yes, n(%)	464 (6.72)	13 (11.93)		477 (6.81)
Education level at 23	n = 6905	n = 109	0.006	n = 7014
Passed A levels, n(%)	1654 (23.95)	18 (16.51)		1672 (23.84)
Passed O levels, n(%)	2884 (41.77)	38 (34.86)		2922 (41.66)
No qualifications, n(%)	2367 (34.28)	53 (48.62)		2420 (34.5)
Social class at 42	n = 6705	n = 97	0.022	n = 6802
Non-manual, n(%)	4380 (65.32)	52 (53.61)		4432 (65.16)
Manual, n(%)	2325 (34.68)	45 (46.39)		2370 (34.84)
Smoking status at 42	n = 7722	n = 129	< 0.001	n = 7851
Non-smoker, n(%)	3962 (51.31)	51 (39.53)		4013 (51.11)
Former smoker, n(%)	1568 (20.31)	18 (13.95)		1586 (20.2)
Smoker: less than 10 cigarettes, n(%)	573 (7.42)	9 (6.98)		582 (7.41)
Smoker: 10–19 cigarettes, n(%)	709 (9.18)	14 (10.85)		723 (9.21)
Smoker: more than 20 cigarettes, n(%)	910 (11.78)	37 (28.68)		947 (12.06)
Alcohol consumption at 42	n = 7723	n = 129	0.198	n = 7852
Moderate, n(%)	3981 (51.55)	59 (45.74)		4040 (51.45)
Abstainers, n(%)	1760 (22.79)	28 (21.71)		1788 (22.77)
Heavy drinking, n(%)	1982 (25.66)	42 (32.56)		2024 (25.78)
Physical activity at 42	n = 7721	n = 129	0.027	n = 7850
Physically active, n(%)	5146 (66.65)	73 (56.59)		5219 (66.48)
Moderately active, n(%)	667 (8.64)	11 (8.53)		678 (8.64)
Inactive, n(%)	1908 (24.71)	45 (34.88)		1953 (24.88)
Housing tenure at 42	n = 7697	n = 128	< 0.001	n = 7825
Own, n(%)	6465 (83.99)	88 (68.75)		6553 (83.74)
Rent from local authority, housing tenure association, n(%)	761 (9.89)	28 (21.88)		789 (10.08)
Rent privately, n(%)	212 (2.75)	5 (3.91)		217 (2.77)
Rent others, n(%)	63 (0.82)	0 (0)		63 (0.81)
Others, n(%)	196 (2.55)	7 (5.47)		203 (2.59)
Cortisol t1	n = 7981	n = 132	0.386	n = 8113
Low, n(%)	6570 (82.32)	113 (85.61)		6683 (82.37)
High, n(%)	1411 (17.68)	19 (14.39)		1430 (17.63)
Cortisol t1-t2	n = 7981	n = 132	0.755	n = 8113
Low, n(%)	6598 (82.67)	111 (84.09)		6709 (82.69)
High, n(%)	1383 (17.33)	21 (15.91)		1404 (17.31)
Fibrinogen	n = 7981	n = 132	< 0.001	n = 8113
Low, n(%)	6133 (76.85)	83 (62.88)		6216 (76.62)
High, n(%)	1848 (23.15)	49 (37.12)		1897 (23.38)
Insulin Growth Factor, IGF-1	n = 7981	n = 132	0.067	n = 8113
Low, n(%)	6244 (78.24)	94 (71.21)		6338 (78.12)
High, n(%)	1737 (21.76)	38 (28.79)		1775 (21.88)
C reactive protein, CRP	n = 7981	n = 132	< 0.001	n = 8113
Low, n(%)	6141 (76.95)	76 (57.58)		6217 (76.63)
High, n(%)	1840 (23.05)	56 (42.42)		1896 (23.37)
Immunoglobulin E, IgE	n = 7981	n = 132	0.351	n = 8113
Low, n(%)	6112 (76.58)	96 (72.73)		6208 (76.52)
High, n(%)	1869 (23.42)	36 (27.27)		1905 (23.48)
Triglycerides	n = 7981	n = 132	0.053	n = 8113
Low, n(%)	6108 (76.53)	91 (68.94)		6199 (76.41)
High, n(%)	1873 (23.47)	41 (31.06)		1914 (23.59)
Ligh Density Lipoprotein, LDL	n = 7981	n = 132	1	n = 8113
Low, n(%)	6261 (78.45)	104 (78.79)		6365 (78.45)
High, n(%)	1720 (21.55)	28 (21.21)		1748 (21.55)
High Density Lipoprotein, HDL	n = 7981	n = 132	0.015	n = 8113
Low, n(%)	6501 (81.46)	96 (72.73)		6597 (81.31)
High, n(%)	1480 (18.54)	36 (27.27)		1516 (18.69)
Glycated haemoglobin, Hb1A1c	n = 7981	n = 132	0.001	n = 8113
Low, n(%)	6374 (79.86)	89 (67.42)		6463 (79.66)
High, n(%)	1607 (20.14)	43 (32.58)		1650 (20.34)
Systolic blood pressure, SBP	n = 7981	n = 132	0.035	n = 8113
Low, n(%)	6046 (75.75)	89 (67.42)		6135 (75.62)
High, n(%)	1935 (24.25)	43 (32.58)		1978 (24.38)
Diastolic blood pressure, DBP	n = 7981	n = 132	0.214	n = 8113
Low, n(%)	6028 (75.53)	93 (70.45)		6121 (75.45)
High, n(%)	1953 (24.47)	39 (29.55)		1992 (24.55)
Peak expiratory flow	n = 7981	n = 132	< 0.001	n = 8113
Low, n(%)	6081 (76.19)	75 (56.82)		6156 (75.88)
High, n(%)	1900 (23.81)	57 (43.18)		1957 (24.12)
Heart rate	n = 7981	n = 132	< 0.001	n = 8113
Low, n(%)	6073 (76.09)	81 (61.36)		6154 (75.85)
High, n(%)	1908 (23.91)	51 (38.64)		1959 (24.15)
Allostatic load	n = 7981	n = 132	< 0.001	n = 8113
Mean (SD)	3.1 (2.1)	4.2 (2.1)		3.1 (2.1)
Allostatic load	n = 7981	n = 132	< 0.001	n = 8113
[0, 3), n(%)	3587 (44.94)	31 (23.48)		3618 (44.6)
[3, 5), n(%)	2574 (32.25)	45 (34.09)		2619 (32.28)
[5, 12], n(%)	1820 (22.8)	56 (42.42)		1876 (23.12)
Neuroendocrine score	n = 7981	n = 132	0.345	n = 8113
0, n(%)	5217 (65.37)	92 (69.7)		5309 (65.44)
[1, 2], n(%)	2764 (34.63)	40 (30.3)		2804 (34.56)
Immune and inflammatory score	n = 7981	n = 132	< 0.001	n = 8113
0, n(%)	3430 (42.98)	39 (29.55)		3469 (42.76)
1, n(%)	2512 (31.47)	32 (24.24)		2544 (31.36)
[2, 4], n(%)	2039 (25.55)	61 (46.21)		2100 (25.88)
Metabolic score	n = 7981	n = 132	0.001	n = 8113
0, n(%)	3600 (45.11)	39 (29.55)		3639 (44.85)
1, n(%)	2607 (32.67)	51 (38.64)		2658 (32.76)
[2,4], n(%)	1774 (22.23)	42 (31.82)		1816 (22.38)
Cardiovascular score	n = 7981	n = 132	< 0.001	n = 8113
0, n(%)	3368 (42.2)	30 (22.73)		3398 (41.88)
1, n(%)	2439 (30.56)	50 (37.88)		2489 (30.68)
[2,4], n(%)	2174 (27.24)	52 (39.39)		2226 (27.44)

Regarding physiological functioning, respondents who died were more likely to have been in the high-risk group for each physiological system with the exception of the neuroendocrine system, and for the following biomarkers: fibrinogen, IGF1, CRP, Triglycerides, HbA1C, heart rate and peak expiratory flow (Table 1). The AL score was higher among participants who died (mean = 4.2,  %low-med-high 23.5–34.1–42.4) than for those who were still alive (mean = 3.1,  %low-med-high 44.9–32.3–22.8).

### Allostatic load and future risk of mortality

**Fig. 1:**
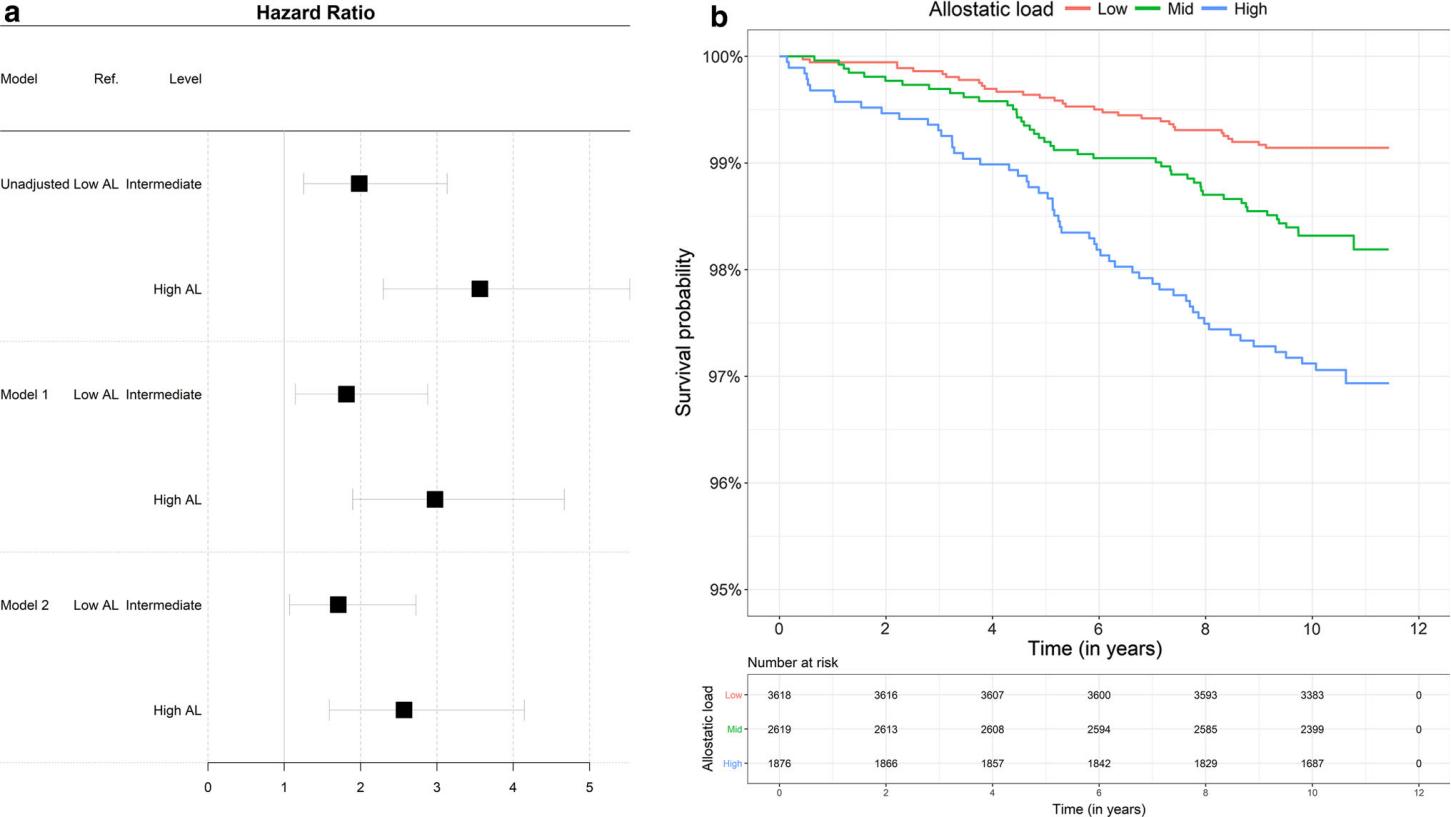
**a** Forest plot of hazard ratio for all-cause of mortality associated with the AL and **b** Kaplan–Meier curve of the Survival probability over the 11 years follow-up period according to category of the allostatic load (N = 8,113, 132 deaths). Allostatic load was classified as low, intermediate, or high as described in Table 2

Multivariable Cox models results are summarised in Table 2 for the imputed data. Hazard ratios for participants with a mid (3 ≤ AL < 5) and high AL (≥ 5) were 1.98 (1.25 to 3.13) and 3.56 (2.2 to 5.53), respectively and were found to be significantly greater than in participants with a low AL (< 3, Crude HR, Table 2, and Fig. 1a). To illustrate the strong association between the AL and future risk of death, the crude survival probability was calculated for the 3 AL groups separately and shows clear differences across AL categories (Fig. 1b). After controlling for early life characteristics (Model 1), adverse childhood experiences and young adulthood risk factors, hazard ratios were 1.81 (1.14 to 2.88) and 2.98 (1.9 to 4.67) for participants with a mid and high AL, respectively, and remained significantly greater than those observed in participants with a low AL. The model 1 effect size estimate of AL on risk of death was slightly attenuated but still significant compared to the crude HR since manual parental social class, smoking heavily during pregnancy, childhood pathologies, and ACEs were significantly associated with higher risk of death (Model 1, Table 2, and Fig. 1a). When, smoking, alcohol, physical activity, BMI, occupation and housing tenure at 42 years old were included in the Cox regression (Model 2), hazard ratios were slightly reduced but still greater for participants with a mid and high AL respectively compared to those with a low AL (1.71 (1.07 to 2.72) and 2.57 (1.59 to 4.15) respectively). Smoking heavily and living in a rented house made a significant contribution to mortality risk (Model 2, Table 2, and Fig. 1a).

**Table 2:** Multivariate Cox proportional hazard regression of all-cause mortality risk for the allostatic load score in 3 groups using data obtained from multiple imputation (N = 8,113)

			Crude hazard ratio		Model 1		Model 2	
Variable	References	Level	HR (95% CI)	*P*-value	HR (95% CI)	*P*-value	HR (95% CI)	*P*-value
Allostatic load	Low AL (0 ≤ AL < 3)	Mid AL (3 ≤ AL < 5)	1.98 [1.25–3.13]	1.7E−03	1.81 [1.14–2.88]	5.7E−03	1.71 [1.07–2.72]	1.2E−02
		High AL (5 ≤ AL ≤ 12)	3.56 [2.3–5.53]	7.0E−09	2.98 [1.9–4.67]	1.1E−06	2.57 [1.59–4.15]	5.6E−05
Sex	Men	Women	0.65 [0.46–0.93]	9.9E−01	0.65 [0.46–0.93]	9.9E−01	0.68 [0.46–1]	9.8E−01
Mother’s education level	Left school at 15 or later	Left school before 14			0.65 [0.42–1]	9.7E−01	0.66 [0.43–1.02]	9.7E−01
Father’s social class at birth	Non-manual	Manual			1.52 [0.91–2.55]	5.2E−02	1.5 [0.89–2.51]	6.2E−02
Overcrowding	1.5 people per room	< 1.5 people per room			1.35 [0.77–2.39]	1.5E−01	1.41 [0.79–2.5]	1.2E−01
Mother’s BMI	Normal	Underweight			0.65 [0.24–1.79]	8.0E−01	0.66 [0.24–1.81]	7.9E−01
		Overweight			1.23 [0.79–1.92]	1.7E−01	1.28 [0.82–2]	1.4E−01
		Obese			1.55 [0.74–3.27]	1.2E−01	1.59 [0.75–3.39]	1.1E−01
Mother smoked during pregnancy	No	Sometimes			1.14 [0.56–2.31]	3.6E−01	1.15 [0.56–2.33]	3.5E−01
		Moderately			1.17 [0.71–1.91]	2.7E−01	1.14 [0.7–1.88]	3.0E−01
		Heavily			1.63 [1.02–2.59]	2.1E−02	1.56 [0.97–2.5]	3.2E−02
Birth weight	Q1: low weight	Q2			0.85 [0.54–1.33]	7.7E−01	0.83 [0.53–1.3]	7.9E−01
		Q3			0.62 [0.37–1.04]	9.6E−01	0.61 [0.36–1.01]	9.7E−01
		Q4:high weight			0.69 [0.4–1.17]	9.2E−01	0.66 [0.39–1.13]	9.4E−01
Childhood pathologies	No	Yes			1.46 [1.01–2.1]	2.1E−02	1.43 [0.99–2.07]	2.7E−02
ACEs	None	One			0.93 [0.59–1.47]	6.2E−01	0.86 [0.54–1.36]	7.4E−01
		Two or more			1.71 [0.99–2.93]	2.6E−02	1.54 [0.89–2.67]	6.0E−02
Malaise inventory at 23	No	Yes			1.37 [0.76–2.47]	1.5E−01	1.22 [0.67–2.23]	2.5E−01
Education level at 23	Passed A levels	Passed O levels			1.01 [0.56–1.81]	4.9E−01	0.93 [0.51–1.69]	6.0E−01
		No qualifications			1.31 [0.73–2.37]	1.8E−01	1.02 [0.53–1.99]	4.7E−01
Social class at 42	Non-manual	Manual					1.13 [0.74–1.73]	2.8E−01
Smoking status at 42	Non-smoker	Former smoker					0.92 [0.54–1.58]	6.2E−01
		Smoker: less than 10 cigarettes					1.19 [0.59–2.43]	3.1E−01
		Smoker: 10–19 cigarettes					1.11 [0.6–2.06]	3.7E−01
		Smoker: more than 20 cigarettes					1.83 [1.14–2.95]	6.5E−03
Alcohol consumption at 42	Moderate	Abstainers					0.74 [0.47–1.19]	8.9E−01
		Heavy drinking					1.11 [0.74–1.68]	3.1E−01
Physical activity at 42	Physically active	Moderately active					1.14 [0.6–2.16]	3.4E−01
		Inactive					1.23 [0.83–1.81]	1.5E−01
BMI at 42	Normal	Underweight					1.69 [0.4–7.04]	2.4E−01
		Overweight					0.79 [0.52–1.2]	8.6E−01
		Obese					1.04 [0.64–1.72]	4.3E−01
House tenure at 42	Own	Rent from local authority, housing tenure association					1.48 [0.91–2.4]	5.6E−02
		Rent privately					1.28 [0.51–3.2]	3.0E−01
		Rent others					NA	NA
		Others					2.05 [0.94–4.47]	3.5E−02

Supplementary Table 4 reports the results of the sensitivity analyses comparing the complete case AL score to the imputed AL score, HR estimates were similar albeit weakened. Additional analyses stratified by gender suggested that the association between high AL and risk of death is equivalent in both sexes (Model 2, in men HR = 2.65 (1.45 to 4.85); in women, HR = 2.48 (1.11 to 5.52); Supplementary Table 5 and 6 respectively).

### Biological systems predictive of the risk of death from all causes

**Fig. 2:**
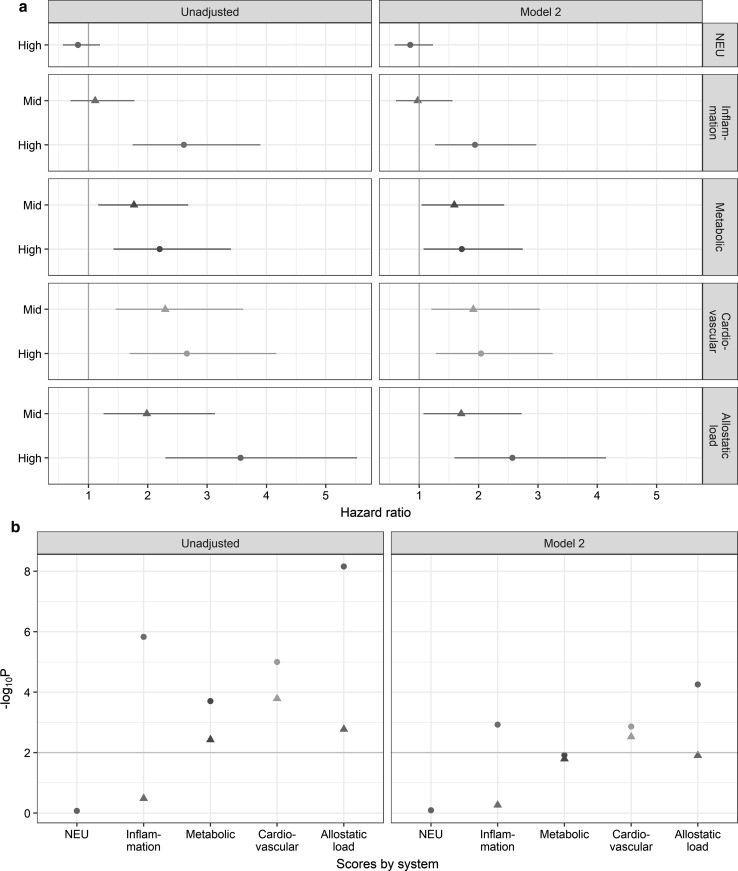
**a** Forest plot of hazard ratio for all-cause of mortality associated with AL and each physiological system and **b** corresponding log10 (*P*-values). The grey line represents a Bonferroni correct *P* value of 0.01. NEU: neuro-endocrine

We examined the impact of the 4 sub-scores representing each physiological system on future risk of death (Table 3). After multiple testing correction (P < 0.01), crude hazard ratios for participants with high sub-score (≥ 5) for all physiological system but the neuro-endocrine were found significantly greater than in participants with a low score (Table 3, Fig. 2). After controlling for early life characteristics, adverse childhood experiences and young adulthood risk factors, being in the high group for the inflammatory, or metabolic, or cardiovascular sub-score was still found to be significantly associated with future risk of death (Model 1, Table 3). When, smoking, alcohol, physical activity, BMI, occupation and housing tenure at 42 years of age were included in the Cox regression (Model 2), only the immune-inflammatory, and cardiovascular sub-score remained significantly related to mortality (Model 2, Table 3, Fig. 2). Irrespective of the physiological system and model, the relationship between the medium class for each sub-score and future risk of death was always weakly associated compared to the high scoring group. In all settings investigated, we observed a greater effect of AL on future risk of death and a weaker signal attenuation after life course adjustment (Table 3, Fig. 2).

**Table 3:** Multivariate Cox proportional hazard regression of all-cause mortality risk for each physiological system of the allostatic load using data obtained from multiple imputation (N = 8113)

			Crude hazard ratio^a^		Model 1^b^		Model 2^c^	
Variable	References	Level	HR (95% CI)	*P*-value	HR (95% CI)	*P*-value	HR (95% CI)	*P*-value
Allostatic load	Low AL (0 ≤ AL < 3)	Mid AL (3 ≤ AL < 5)	1.98 [1.25–3.13]	1.69E−03	1.81 [1.14–2.88]	5.67E−03	1.71 [1.07–2.72]	1.24E−02
		High AL (5 ≤ AL ≤ 12)	3.56 [2.3–5.53]	6.95E−09	2.98 [1.9–4.67]	1.05E−06	2.57 [1.59–4.15]	5.57E−05
Neuroendocrine score	Low (0)	High (≥ 1)	0.82 [0.57–1.19]	8.47E−01	0.82 [0.57–1.19]	8.47E−01	0.85 [0.58–1.23]	8.06E−01
Immune and inflammatory score	Low (0)	Mid (1)	1.11 [0.7–1.77]	3.28E−01	1.06 [0.66–1.69]	4.10E−01	0.97 [0.61–1.56]	5.45E−01
		High (≥ 2)	2.61 [1.74–3.9]	1.48E−06	2.25 [1.5–3.39]	5.05E−05	1.94 [1.26–2.97]	1.20E−03
Metabolic score	Low (0)	Mid (1)	1.77 [1.16–2.68]	3.74E−03	1.61 [1.06–2.45]	1.29E−02	1.59 [1.04–2.43]	1.61E−02
		High (≥ 2)	2.2 [1.42–3.4]	1.97E−04	1.86 [1.19–2.9]	3.00E−03	1.72 [1.07–2.74]	1.21E−02
Cardiovascular score	Low (0)	Mid (1)	2.29 [1.46–3.61]	1.63E−04	2.14 [1.35–3.37]	5.63E−04	1.91 [1.2–3.03]	3.00E−03
		High (≥ 2)	2.66 [1.7–4.17]	1.01E−05	2.36 [1.5–3.72]	1.05E−04	2.04 [1.28–3.25]	1.37E−03

^a^Sex-adjusted hazard ratio

^b^Adjusted for early life, ACEs, behaviours at 23

^c^Model 1 + behaviours at 42

**Fig. 3:**
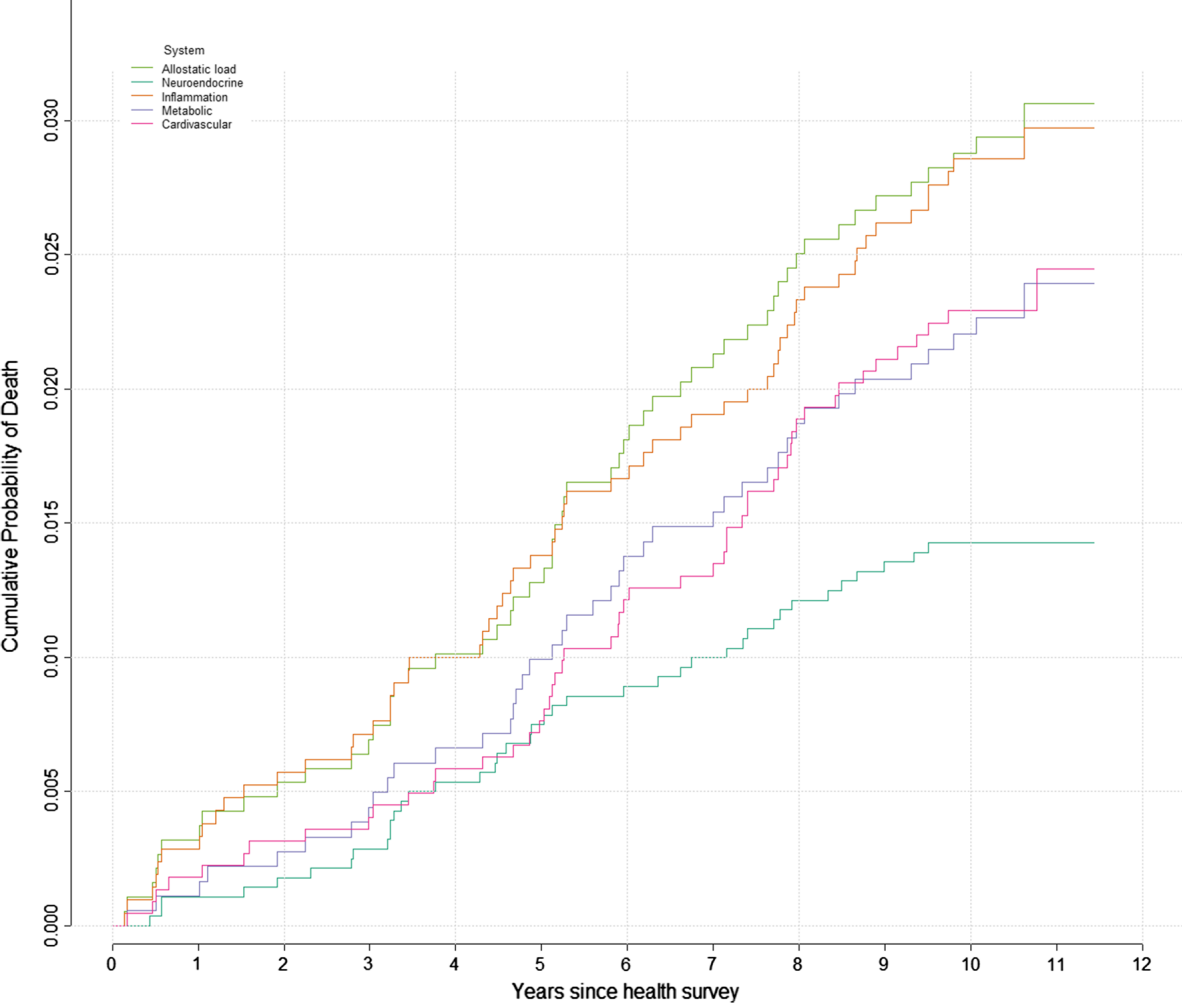
Kaplan-Meier probability of the cumulative probability of death according to each physiological system and the AL. Cumulative mortality is shown for the ‘high’ AL score and each ‘high’ physiological sub-score

As depicted on Fig. 3, the inflammatory/immune curve is the closest to the AL curve followed by the cardiovascular and metabolic curves suggesting a prominent role of inflammation with future risk of death between 44 and 55 years of age. However participants with high AL had a greater risk of death compared to each physiological systems.

### Biomarkers predictive of the risk of death from all causes

**Fig. 4:**
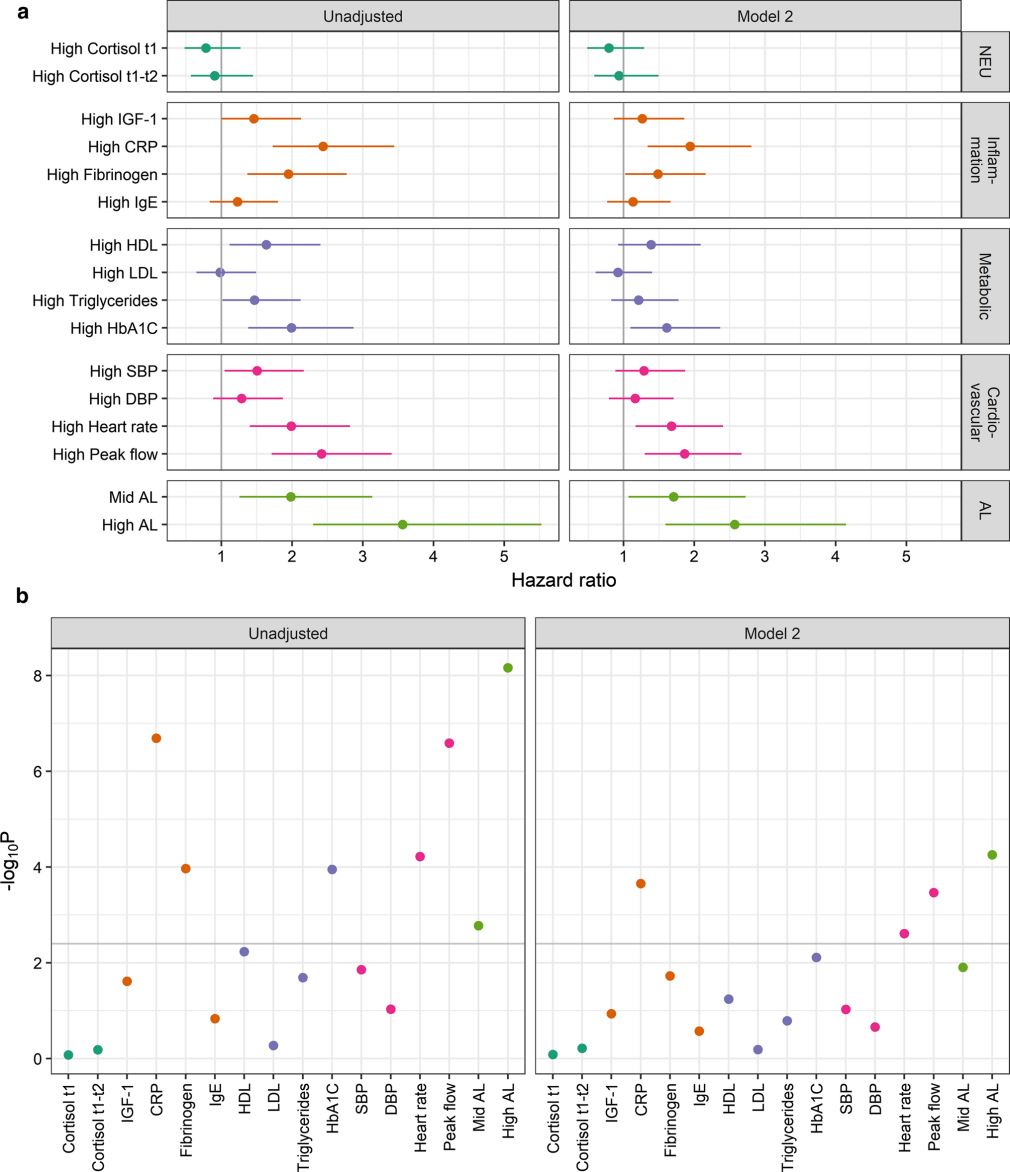
**a** Forest plot of hazard ratio for all-cause of mortality associated with AL and each physiological biomarkers grouped by system and **b** corresponding log10 (*P*-values). The grey line represents a Bonferroni correct P-value of 0.003. NEU: neuro-endocrine. AL: allostatic load

In a final set of analyses, we examined the relative individual contributions of each of the 14 biomarkers constituting our AL index to the risk of mortality. Analyses reported in Table 4 show the impact of each individual biological measure considered separately on mortality in an unadjusted model, and then gradually adjustments are made (model 1 and model 2 comparable to models in Table 2). After multiple testing correction (P < 0.003) and adjustment for early life characteristics, adverse childhood experiences and young adulthood risk factors, five biomarkers (CRP, fibrinogen, glycated haemoglobin, heart rate and peak expiratory flow) from three physiological system (inflammatory and immune, metabolic and cardiovascular system) were significantly related to mortality (Model 1, Table 4). Additional adjustment for smoking, alcohol, physical activity, BMI, occupation and housing tenure at 42 years old resulted in greater risks only for participants a high CRP, fibrinogen, heart rate and peak flow compared to those with a low score (Model 2, Table 4, Fig. 4).

**Table 4:** Multivariate Cox proportional hazard regression of all-cause mortality risk for individual biological components of the allostatic load using data obtained from multiple imputation (N = 8113)

			Crude hazard ratio^a^		Model 1^b^		Model 2^c^	
Variable	References	Level	HR (95% CI)	*P*-value	HR (95% CI)	*P*-value	HR (95% CI)	*P*-value
Allostatic load	Low AL (0 ≤ AL < 3)	Mid AL (3 ≤ AL < 5)	1.98 [1.25–3.13]	1.69E−03	1.81 [1.14–2.88]	5.67E−03	1.71 [1.07–2.72]	1.24E−02
		High AL (5 ≤ AL ≤ 12)	3.56 [2.3–5.53]	6.95E−09	2.98 [1.9–4.67]	1.05E−06	2.57 [1.59–4.15]	5.57E−05
Neuroendocrine system								
Cortisol t1	Low (0)	High (1)	0.78 [0.48–1.27]	8.40E−01	0.79 [0.48–1.28]	8.31E−01	0.79 [0.49–1.29]	8.25E−01
Cortisol t1-t2	Low (0)	High (1)	0.91 [0.57–1.45]	6.58E−01	0.9 [0.56–1.43]	6.75E−01	0.94 [0.59–1.5]	6.10E−01
Immune and inflammatory system								
Insulin Growth Factor, IGF-1	Low (0)	High (1)	1.46 [1–2.13]	2.45E−02	1.39 [0.95–2.03]	4.43E−02	1.26 [0.86–1.86]	1.16E−01
C reactive protein, CRP	Low (0)	High (1)	2.44 [1.73–3.45]	2.05E−07	2.14 [1.51–3.04]	1.03E−05	1.94 [1.34–2.81]	2.22E−04
Fibrinogen	Low (0)	High (1)	1.95 [1.37–2.77]	1.07E−04	1.69 [1.18–2.42]	2.09E−03	1.49 [1.02–2.16]	1.88E−02
Immunoglobulin E, IgE	Low (0)	High (1)	1.23 [0.84–1.8]	1.46E−01	1.2 [0.81–1.76]	1.80E−01	1.13 [0.77–1.66]	2.66E−01
Metabolic system								
High Density Lipoprotein, HDL	Low (0)	High (1)	1.64 [1.12–2.4]	5.85E−03	1.44 [0.97–2.12]	3.38E−02	1.39 [0.92–2.09]	5.71E−02
Light Density Lipoprotein, LDL	Low (0)	High (1)	0.98 [0.65–1.49]	5.32E−01	0.96 [0.63-1.46]	5.80E−01	0.92 [0.6-1.4]	6.50E−01
Triglycerides	Low (0)	High (1)	1.47 [1.02–2.12]	2.05E−02	1.31 [0.9–1.9]	7.79E−02	1.21 [0.83–1.78]	1.62E−01
Glycated haemoglobin, Hb1A1c	Low (0)	High (1)	1.99 [1.38–2.87]	1.12E−04	1.76 [1.22–2.55]	1.34E−03	1.61 [1.09–2.37]	7.75E−03
Cardiovascular system								
Systolic blood pressure, SBP	Low (0)	High (1)	1.5 [1.05–2.16]	1.40E−02	1.42 [0.98–2.05]	3.05E−02	1.29 [0.88–1.87]	9.41E−02
Diastolic blood pressure, DBP	Low (0)	High (1)	1.29 [0.89–1.87]	9.32E−02	1.23 [0.84–1.79]	1.40E−01	1.16 [0.79–1.71]	2.19E−01
Heart rate	Low (0)	High (1)	1.99 [1.4–2.82]	6.06E−05	1.91 [1.34–2.72]	1.63E−04	1.68 [1.17–2.41]	2.46E−03
Peak expiratory flow	Low (0)	High (1)	2.42 [1.71–3.41]	2.58E−07	2.09 [1.47–2.97]	2.16E−05	1.86 [1.3–2.67]	3.44E−04

^a^Sex-adjusted hazard ratio

^b^Adjusted for early life, ACEs, behaviours at 23

^c^Model 1 + behaviours at 42

**Fig. 5:**
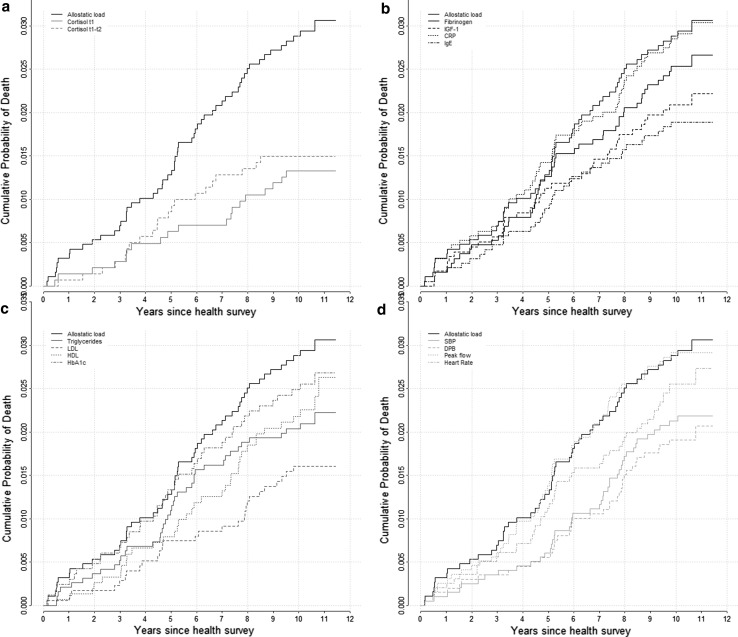
Kaplan-Meier probability of the cumulative probability of death according to each individual biomarker and the AL. Cumulative mortality is shown for the ‘high’ AL score and each ‘high’ individual components: neuroendocrine (**a**), immune and inflammatory (**b**), metabolic system (**c**) and cardiovascular (**d**)

Figure 5 shows the cumulative probability of death for persons with high individual biomarkers and high AL by physiological system. For the inflammatory/immune system, the probability of death was slightly worse for participants with a high AL compared to a high CRP level (Fig. 5a) or a high peak flow (Fig. 5b).

Our results at the biomarker level were consistent with the results at the physiological system level; the inflammatory/immune and cardiovascular systems each containing the more influential biomarkers, notably CPR and peak flow and to lesser extent fibrinogen and heart rate.

## Discussion

Our study suggests that having a higher allostatic load score was significantly associated with an increased risk of all-cause premature mortality over an 11 year period. After controlling for early life SEP, other life course factors and health status, participants with a high AL at 44 years old had a risk of dying before 55y almost 3 times higher as those with a low AL providing evidence that the observed association was independent of early life SEP and other life course factors. Adding SEP and health status at 42 slightly attenuated this association [HR = 2.57 (1.59 to 4.15)], with heavy smoking and living in rented accommodation making a significant contribution to mortality risk.

We also analysed the relationship between the components of our AL score and mortality, examining to what extent each system or individual biomarker contributed to the increased risk of death. Among the four physiological systems composing our AL score, only the immune-inflammatory and cardiovascular sub-scores remained significantly related to mortality after adjustment and multiple testing correction suggesting a pivotal role of these systems in our allostatic load index. To evaluate the contribution of each sub-score in the relationship between AL and future risk of death, additional analyses controlling for each physiological system were performed (Supplementary Table 7). Adding either the inflammatory/immune or the cardiovascular sub-score had the greater effect on the AL HR estimate. Nevertheless the association between AL and future risk of death was only slightly weakened HR = 2.17 (1.22 to 3.89) and HR = 2.22 (1.27 to 3.88) after controlling respectively for the inflammatory/immune and cardiovascular sub-score. Participants with a high CRP, fibrinogen, heart rate and peak flow had a greater risk of death compared to those with a low score. Similarly when controlling for CRP, fibrinogen, heart rate and peak flow in the relationship between AL and future risk of death, the association was mostly affected by CRP [HR = 2.27 (1.36 to 3.68)] followed by peak flow [HR = 2.26 (1.38 to 3.68)] and heart rate [HR = 2.27 (1.36 to 3.77)] and to less extent fibrinogen [HR = 2.42 (1.46 to 4.03), Supplementary Table 8]. Our results suggest that the cumulative AL measure consisting of all the biomarkers was a better measure for predicting death compared to each sub-score and biomarker analysed separately, in line with studies in US [28, 32–35], Taiwan [36] and Scotland [37], and with the assumption of global physiological wear and tear captured by AL.

Our findings should be understood in the light of our previous research which highlighted the pathways between a lower socioeconomic position at birth and having a higher allostatic load at age 45 in the same cohort study [22]. We showed that the most important indirect pathway, explaining up to 31% of the effect, was through the cohort members’ educational attainment. However, the majority of the association (up to 68%) between early life social position and mid-life allostatic load was unexplained by any of the mediating factors. We also previously analysed the pathways between adverse childhood conditions and allostatic load, showing that the association operated largely via education, health behaviours, wealth and BMI in adulthood [23]. Here, our work examines the other end of the life course, focusing on the association between allostatic load and mortality before 55 years of age. Our findings show that the association between allostatic load in mid-life, and mortality is virtually unchanged after taking into account many of the major early life factors, including socioeconomic position at birth and adverse childhood experiences, and after including mediating factors such as health behaviours, socioeconomic position and housing tenure in adulthood. This suggests that the observed relationship between such an indicator of multi-system physiological wear-and-tear in mid-life and death is largely independent in this cohort. We suggest therefore that biological risk, and notably degraded inflammatory (through CRP and fibrinogen) and cardiovascular (through peak flow and heart rate) systems, are driving the association with the specific types of mortality observed at this early stage of the life course. Unfortunately, cause-specific mortality is currently unavailable for the cohort, which would allow us to examine this further. These findings highlight the potential importance of biological risk scores, such as allostatic load, in capturing both pre-disease states and the social embedding.

Several limitations of this study should be considered. Since analyses were performed using a birth cohort, an important weakness is related to attrition and selection bias. However, the surviving cohort remains broadly representative of the initial cohort on key childhood and adult characteristics [27]. To allow for uncertainty about the missing data and to ensure distribution, variability, and relationships between variables, multiple imputations were used for confounding variables taking the missing at random assumption. In addition, we ran all analyses on complete cases i.e. individuals with no missing data for any of the selected characteristics. The smaller sample size reduced statistical power, and measures of association were subsequently weakened. Nevertheless, the hazard ratios were all consistent with the imputed results. Given the small number of deaths included in our study (N = 132), and that dead participants who were excluded (no blood collected, N = 234) were most likely to come from overcrowded households and their mothers were more likely to have left school before 14 years old (Supplementary Table 4), and considering the negative association between mother’s education level and AL, our results might be conservative. We cannot exclude the possibility that other factors may contribute to the mechanisms linking AL and future risk of death. Since AL is a latent variable capturing multi-system physiological wear-and-tear and there is no standard measure for capturing it, another limitation is in the measurement of AL, which varies across studies. We used 14 available biological parameters representing four physiological systems. However, we were constrained by variable availability and lack ‘primary’ biomarkers (epinephrine and norepinephrine), which means that the neuroendocrine system was poorly represented compared to the others [38]. There is currently no consensus regarding (i) the choice of the relevant markers to be included (ii) their measurement (iii) their combination and (iv) ad-hoc statistical analyses [15, 16, 39]. Furthermore, the relative importance of each AL component in the stress response cascade remains to be explored in order to better capture physiological wear-and-tear.

Despite the limitations mentioned above, we used a longitudinal population-based birth cohort collecting data prospectively across the life span. Important strengths include: the large sample size for the biomedical survey; the large number of biomarkers available; the great detail and breadth of variables within the cohort which allowed us to control for a number of potential confounding factors.

Multiple studies have addressed the relationship between AL and future risk of death. Higher allostatic load was a significant predictor of functional decline [40] and mortality across different time periods and countries [28, 32–37], a relationship not attributable to age, sex, ethnicity, education, or income. Seeman et al. used data from the MacArthur Successful Aging Study, a longitudinal study of men and women aged 70-79 living in the United States and reported that higher baseline AL scores were associated with significantly greater risk for mortality within 7 years [28]. Karlamangla et al. further examined the effect of changes in AL on mortality risk using two measures of AL on a smaller sample, they reported that participants whose AL increased had higher risk of all-cause mortality [32]. Using data from the third National Health and Nutrition Examination Survey (NHANES III) (1988–1994), a large, nationally representative study of around 40,000 U.S. children and adults, Borrell et al. found an increased risk of all-cause mortality within 12 years in participants with AL scores of two, three and above compared to those with an AL score of zero or one, independently of ethnicity, income and education [33]. Levine & Crimmins found that participants in the top AL quintile had higher overall mortality risk within 10 years compared to participants in the lowest AL quintile after adjusting for age and sex [34]. Howard and Sparks showed that each 1-point increase in AL was associated with 7% incremental risk of mortality controlling for age, sex, ethnicity, SEP and health behaviours [35]. Hwang et al. used the Taiwanese Social Environment and Biomarkers of Aging Study, a longitudinal survey of adults 54 years and older, reported that a higher AL was significantly associated with 10-year increased risk of death [36]. More recently, Robertson et al. used data from the Scottish Health Survey and identified increased risk of all-cause mortality within 10 years associated with increasing AL [37]. Our study fits into this international literature, with the added strength of being a birth cohort where the relationship between variables over time facilitates our understanding of underlying mechanisms. We also focused on understanding the drivers behind the relationship between AL and subsequent mortality by examining physiological sub-scores. We found that the relationship was notably driven by two major systems: the immune-inflammatory and cardiovascular system. Specifically, four individual biomarkers were identified as driving the association: CRP, fibrinogen, for the immune-inflammatory system; heart rate and peak flow for the cardiovascular system. Our results were consistent with cause of death registered in England and Wales in 2013 (Office for National Statistics (www.ons.gov.uk)) where neoplasms (notably lung cancers) were the first cause of death in both men and women between 50-59 years old followed by diseases of the cardiovascular system. Our study suggests that the relationship between AL, its components and future risk of death at 55 years may be a reflection of both age and cause-specific nature of death at this stage of the life course. These findings may provide evidence that immune-inflammatory and cardiovascular wear-and-tear remain areas for primary prevention at earlier phases of the life course, given their importance in driving premature mortality risk and subsequent ageing patterns.

Since biomarker information was only available at 44 years of age, the AL measurement we used does not provide information on dynamic change over time and does not fully capture the flexibility of stress response mechanisms across the lifespan. Our findings support the conceptual validity of AL as being able to provide insight into cumulative risks to health across multiple physiological system [28].

To take into account the complexity and the dynamic nature of AL as the result of adaptation to environmental challenges, additional studies are required to define a set of representative physiological systems, identify time-specific biomarkers and investigate AL at multiple time points in population longitudinal studies across contexts. Strategies to increase physiological resilience along with targeted prevention policies over the life course to manage exposures and physiological responses to stress are necessary to prevent its detrimental effects on health.

## Electronic supplementary material

Below is the link to the electronic supplementary material.

Supplementary material 1 (XLSX 42 kb)
